# Supplementary material for: COZOID: contact zone identifier for visual analysis of protein-protein interactions
Source: BMC Bioinformatics. 2018 Apr 6;19:125. doi: 10.1186/s12859-018-2113-6 (PMC5889581; doi:10.1186/s12859-018-2113-6)
Supplement: Supplementary file 2 — Software build. Executable binary file of the software tool. (ZIP 118,784 kb) [file 12859_2018_2113_MOESM2_ESM.zip › cozoid/java/docs/beansbinding-1.2.1-doc.zip!/org/jdesktop/swingbinding/package-summary.html]

org.jdesktop.swingbinding (Beans Binding)


---


|  |  |  |
| --- | --- | --- |
| **Overview** \| **Package** \| Class \| **Use** \| **Tree** \| **Deprecated** \| **Index** \| **Help** | |  |
| **PREV PACKAGE**   **NEXT PACKAGE** | **FRAMES**    **NO FRAMES**     **All Classes** |  |


---

## Package org.jdesktop.swingbinding

Provides support for binding to complex Swing components, and
documentation on the interesting Swing properties to bind to.

**See:**
  
          **Description**

| **Class Summary** | |
| --- | --- |
| **JComboBoxBinding<E,SS,TS>** | Binds a `List` of objects to act as the items of a `JComboBox`. |
| **JListBinding<E,SS,TS>** | Binds a `List` of objects to act as the elements of a `JList`. |
| **JTableBinding<E,SS,TS>** | Binds a `List` of objects to act as the rows of a `JTable`. |
| **SwingBindings** | A factory class for creating instances of the custom Swing `Binding` implementations provided by this package. |

## Package org.jdesktop.swingbinding Description

Provides support for binding to complex Swing components, and
documentation on the interesting Swing properties to bind to.

### Interesting Swing Properties

Any Swing component property that conforms to the Java Beans specification
is an excellent candidate for use by `BeanProperty`, `ELProperty`
and other `Property` implementations that resolve properties in a
similar manner. In addition, adapters have been pre-registered for a handful
of properties that don't correctly conform to the specification
(in this case, don't fire property change notificiation), and a handful
of synthetic properties, so that they can be used in the same way.
The complete list of adapted and synthetic properties is below:

| Component | Property | Description |
| --- | --- | --- |
| **`AbstractButton`** | `"selected"` | The selected state of a button. |
|  | | |
| **`JComboBox`** | `"selectedItem"` | The selected item of a `JComboBox`. |
|  | | |
| **`JSpinner`** | `"value"` | The value of a `JSpinner`. |
|  | | |
| **`JSlider`** | `"value"` | The value of a `JSlider`; notifies of all changes. |
|  | `"value_IGNORE_ADJUSTING"` | Same as `"value"` but does not notify of change while the slider is adjusting its value. |
|  | | |
| **`JList`** | `"selectedElement"`   (Currently read-only) | The selected element of a `JList`; notifies of all changes. If there is a `JListBinding` with the `JList` as the target, the selected element is reported as an element from the binding's source list. Otherwise, the selected element is reported as an object from the list's model. If nothing is selected, the property evaluates to `null`. |
|  | `"selectedElements"`   (Currently read-only) | A list containing the selected elements of a `JList`; notifies of all changes. If there is a `JListBinding` with the `JList` as the target, the selected elements are reported as elements from the binding's source list. Otherwise, the selected elements are reported as objects from the list's model. If nothing is selected, the property evaluates to an empty list. |
|  | `"selectedElement_IGNORE_ADJUSTING"`   (Currently read-only) | Same as "selectedElement" but does not notify of change while the list selection is being updated. |
|  | `"selectedElements_IGNORE_ADJUSTING"`   (Currently read-only) | Same as "selectedElements" but does not notify of change while the list selection is being updated. |
|  | | |
| **`JTable`** | `"selectedElement"`   (Currently read-only) | The selected element of a `JTable`; notifies of all changes. If there is a `JTableBinding` with the `JTable` as the target, the selected element is reported as an element from the binding's source list. Otherwise, the selected element is reported as a map where the keys are composed of the string "column" plus the column index and the values are the model values for that column. Example: `{column0=column0value, column1=column1value, ...}` If nothing is selected, the property evaluates to `null`. |
|  | `"selectedElements"`   (Currently read-only) | A list containing the selected elements of a `JTable`; notifies of all changes. If there is a `JTableBinding` with the `JTable` as the target, the selected elements are reported as elements from the binding's source list. Otherwise, each selected element is reported as a map where the keys are composed of the string "column" plus the column index and the values are the model values for that column. Example: `{column0=column0value, column1=column1value, ...}` If nothing is selected, the property evaluates to an empty list. |
|  | `"selectedElement_IGNORE_ADJUSTING"`   (Currently read-only) | Same as "selectedElement" but does notify of change while the table selection is being updated. |
|  | `"selectedElements_IGNORE_ADJUSTING"`   (Currently read-only) | Same as "selectedElements" but does not notify of change while the table selection is being updated. |
|  | | |
| **`JTextComponent`** | `"text"` | The text property of a `JTextComponent`; notifies of all changes (including typing). |
|  | `"text_ON_FOCUS_LOST"` | The text property of a `JTextComponent`; notifies of change only when focus is lost on the component. |
|  | `"text_ON_ACTION_OR_FOCUS_LOST"` | The text property of a `JTextComponent`; notifies of change only when the component notifies of `actionPerformed` or when focus is lost on the component. |

### Swing Binding Subclasses

This package supports binding to the more complex Swing components by providing
custom `Binding` subclasses tailored to the needs of these components.
These subclasses are:

| Binding class | Description |
| --- | --- |
| **`JComboBoxBinding`** | Bind a `java.util.List` of items to be used as the items in a `JComboBox`. |
| **`JListBinding`** | Bind a `java.util.List` of elements to be the elements of a `JList`, and specify how the elements are displayed. |
| **`JTableBinding`** | Bind a `java.util.List` of elements to be the elements of a `JTable`, and specify how properties of the elements are mapped to columns. |
| **more to come...** | |

Instances of these classes are obtained by invoking the static `create`
methods provided by the `SwingBindings` class.

### Examples

Bind a list of `Person` beans to a `JTable` and have it show
the `"firstName"` property of each bean in column 0 and the `"lastName"`
property in column 1:

```
    // create the person list
    List people = createPersonList();
            
    // create the binding from list to table
    JTableBinding tb = SwingBindings.createJTableBinding(READ, people, table);

    // configure how the properties map to columns
    tb.addColumnBinding(BeanProperty.create("firstName"));
    tb.addColumnBinding(BeanProperty.create("lastName"));

    // realize the binding
    tb.bind();
```

Given the binding above, create a second set of bindings that bind
the `"firstName"` property of the table's selected element to one
`JTextField` and the `"lastName"` property of the selected
element to another. Furthermore, specify that we want the text fields to
report change (and therefore have their values committed back to the
selected element) only on when focus is lost from the field:

```
    // create properties representing the selected person's first name and last name
    Property selectedFirstP = BeanProperty.create("selectedElement.firstName");
    Property selectedLastP = BeanProperty.create("selectedElement.lastName");

    // create a property representing a text field's text with change reported only on focus lost
    Property textP = BeanProperty.create("text_ON_FOCUS_LOST");

    // bind the selected first name and last name to the two text fields
    Binding b1 = Bindings.createAutoBinding(READ_WRITE, table, selectedFirstP, field1, textP);
    Binding b2 = Bindings.createAutoBinding(READ_WRITE, table, selectedLastP, field2, textP);
    
    // realize the bindings
    b1.bind();
    b2.bind();
```

What you've seen above is actually the simple building blocks of a typical master-detail application.

---


|  |  |  |
| --- | --- | --- |
| **Overview** \| **Package** \| Class \| **Use** \| **Tree** \| **Deprecated** \| **Index** \| **Help** | |  |
| **PREV PACKAGE**   **NEXT PACKAGE** | **FRAMES**    **NO FRAMES**     **All Classes** |  |


---
